# Placental thrombosis in acute phase abortions during experimental *Toxoplasma gondii* infection in sheep

**DOI:** 10.1186/1297-9716-45-9

**Published:** 2014-01-29

**Authors:** Pablo Castaño, Miguel Fuertes, Ignacio Ferre, Miguel Fernández, Maria del Carmen Ferreras, Javier Moreno-Gonzalo, Camino González-Lanza, Frank Katzer, Javier Regidor-Cerrillo, Luis Miguel Ortega-Mora, Valentín Pérez, Julio Benavides

**Affiliations:** 1 Departamento de Sanidad Animal, Instituto de Ganadería de Montaña (CSIC-ULE), Grulleros, 24346 León, Spain; 2 SALUVET, Animal Health Department, Faculty of Veterinary Sciences, Complutense University of Madrid, Ciudad Universitaria s/n, 28040 Madrid, Spain; 3 Moredun Research Institute, Pentlands Science Park, Bush Loan, Edinburgh EH26 0PZ, UK

## Abstract

After oral administration of ewes during mid gestation with 2000 freshly prepared sporulated oocysts of *T. gondii* isolate M4, abortions occurred between days 7 and 11 in 91.6% of pregnant and infected ewes. Afterwards, a further infection was carried out at late gestation in another group of sheep with 500 sporulated oocysts. Abortions happened again between days 9 and 11 post infection (pi) in 58.3% of the infected ewes. Classically, abortions in natural and experimental ovine toxoplasmosis usually occur one month after infection. Few experimental studies have reported the so-called acute phase abortions as early as 7 to 14 days after oral inoculation of oocysts, and pyrexia was proposed to be responsible for abortion, although the underline mechanism was not elucidated. In the present study, all placentas analysed from ewes suffering acute phase abortions showed infarcts and thrombosis in the caruncullar villi of the placentomes and ischemic lesions (periventricular leukomalacia) in the brain of some foetuses. The parasite was identified by PCR in samples from some placentomes of only one sheep, and no antigen was detected by immunohistochemical labelling. These findings suggest that the vascular lesions found in the placenta, and the consequent hypoxic damage to the foetus, could be associated to the occurrence of acute phase abortions. Although the pathogenesis of these lesions remains to be determined, the infectious dose or virulence of the isolate may play a role in their development.

## Introduction

Ovine toxoplasmosis is a zoonotic disease of sheep caused by the infection of the protozoan *Toxoplasma gondii* that results in heavy economic losses for the sheep industry as it is a major cause for reproductive failure. High seroprevalence of infection has been worldwide reported, and it is recognised as one of the main ovine infectious abortifacient in those countries with a significant sheep industry [1]. Congenital transmission to the foetus occurs mainly when sheep are infected for the first time during pregnancy and the outcome of the infection depends on the gestational age at the time of transplacental transmission. Infections during the early and mid pregnancy are usually associated with the occurrence of abortion, while infection in late pregnancy would produce a congenitally infected but viable lamb [2].

Despite the great importance of this disease, for both animals and public health, the cause of abortion in toxoplasmosisis not yet fully understood [3]. Early foetal death is thought to be caused by the direct damage of the parasite replication in the foetus, while foetal death later in gestation may result from anoxia caused by extensive necrosis in the placentomes [4]. Lesions in the placenta could appear as soon as 10 days after infection, and are characterized by multiple small foci of necrosis in the caruncular septa. Lesions in the foetus could be found from day 15 post infection (pi), although are more frequent from day 20, affecting the brain, heart, lung and liver. They are characterized by multiple foci of necrosis with an eosinophilic central area that can be surrounded by glial cells in the brain or mononuclear inflammatory cells elsewhere when infection occurs at mid or late gestation [5]. Parasite can be identified in relation to microscopic lesions by immunohistochemical labelling of histological slides or PCR amplification of *T. gondii*[6].

Once the infection occurs, there is generally a delay of 4 weeks until the occurrence of the abortion [3]. However, in a number of experimental studies, oral inoculation of sheep with sporulated oocysts of *T. gondii* resulted in an earlier abortion between days 7 and 14 pi. For such presentation of ovine toxoplasmosis, the term “acute phase abortion” was coined [7,8]. In these cases, the parasite was not found in placental or foetal samples and the ewes did not show serological antibodies against the parasite at the time of abortion, although these were detected few days after [8]. No histological examination of the placenta or foetus was carried out in these cases so the absence of detectable parasite in them led the authors to propose that the pyrexia developed after the infection was the cause of abortion, although the exact mechanisms involved were not elucidated [7,8]. Furthermore, the authors raised the hypothesis that the occurrence of acute phase abortions related to toxoplasmosis may be seriously under diagnosed in the field due to the lack of antibody response and parasite detection [7]. It was estimated that 1 out of every 7 abortion associated with toxoplasmosis could be acute phase abortions [8].

In the current study we report the occurrence of acute phase abortions during an experimental infection of sheep with sporulated oocysts of *T. gondii* at mid and late gestation. In addition, in these foetuses and their dams, clinical, histopathological, PCR and immunological studies were carried out in order to investigate the pathogenesis of this clinical presentation of ovine toxoplasmosis.

## Materials and methods

### Inoculum preparation

Sporulated oocysts were obtained through oral infection of cats according to the methods proposed elsewhere [9]. Briefly, ten 8 week-old female Balb/c mice (Charles River Laboratories, France) were inoculated intra peritoneally with 10^4^ tachyzoites of the M4 isolate of *T. gondii* (Moredun Research Institute, Edinburgh, Scotland, UK) suspended in 200 μL PBS. All infected mice were treated with sulphadimidine sodium (0.3 mg/mL in drinking water) for 14 days, beginning 10 days post-infection (dpi), to minimize morbidity and prevent death. At 4 months post-inoculation mice were humanely euthanized by CO_2_ asphyxiation and brains were removed from each mouse. Two twelve-week-old specific-pathogen-free kittens (Isoquimen S.L., Barcelona, Spain) were fed a pool of 3 brains each from the infected mice.

Faeces were collected from kittens daily and examined by zinc sulfate double centrifugation to detect shedding of *T. gondii* oocysts as well as to monitor a possible co-infection with other parasites. Unsporulated oocysts were harvested from faeces using a saturated sodium chloride solution to concentrate them by flotation [10]. Oocysts were counted on a hemocytometer. Oocysts were sporulated by resuspending on H_2_SO_4_ 2% for 4 days at room temperature. Sporulated oocysts were kept at 4 °C until used. All procedures were conducted in a biohazard hood.

### Animals and experimental design

Seventy pure Churra breed sheep aged approximately 12 months (born at the same flock within the same lambing season from synchronized ewes), seronegative for *T. gondii*, *Neospora caninum,* Border disease virus, *Coxiella burnetii* and *Chlamydophila abortus* were oestrus synchronized and mated with pure breed Churra tups for 2 days, after which the rams were removed from the ewes. Pregnancy and foetal viability were confirmed by ultrasound scanning on day 40 after mating and forty-eight pregnant sheep were selected for the experiment. The animals were randomly allocated into two groups: A and B. Each group was composed of twenty-four animals: sixteen sheep to be infected and eight uninfected-control animals. Animals were observed by a veterinarian twice daily throughout the experiment.

Each group was independently allocated for two different experimental infections. For the first infection, sixteen animals from group A were each inoculated with 2 × 10^3^ sporulated oocysts diluted in 50 mL of PBS, orally administered in a single dose, on day 90 of gestation. Oocysts were used one month after sporulation. For the second infection, carried out thirty days later, sixteen sheep from group B each inoculated at day 120 of pregnancy with a single oral dose of 5 × 10^2^ sporulated oocysts from the same batch as used before. In each group, eight sheep received 50 mL of PBS without oocysts as negative control of infection. Rectal temperatures were recorded from group B animals two days before inoculation and then daily until 14 dpi.

Initial design of the experiment involved the serial culling of four infected and two uninfected-control animals at 5, 12, 21 and 26 dpi by intravenous barbiturate overdose.

All animal procedures complied with the Guidelines of the European Union Council (2010/63/EU) for the use of laboratory animals and were in accordance with local national guidelines (RD 1201/2005) which regulates the welfare of animals used for experimentation. They were also approved by the Complutense University of Madrid and CSIC bioethics committee.

### Histological processing and immunohistochemical labelling of tissues

Post-mortem examinations of all the culled ewes and foetuses recovered were carried out immediately after euthanasia (or abortion in a number of cases. See Results section) and tissue samples were taken and placed into 10% formaldehyde in buffered saline. From each placenta, ten randomly selected placentomes were chosen and sliced coronally. Maternal samples included brain and iliofemoral (uterine) and mesenteric caudal lymph nodes. Foetal tissue samples included brain, spinal cord, apex of the heart, lung, liver, kidney, thymus and semitendinosus muscle. After fixation for less than five days, maternal and foetal brains were cut coronally, embedded in paraffin wax and processed, with the rest of the samples, by standard procedures for haematoxylin and eosin staining. Selected sections from the placentomes were stained with Martius, Scarlet and Blue (MSB) for the detection of fibrin.

After microscopic examination, selected tissue sections from the placental and foetal brain samples of all animals were immunolabelled for *T. gondii* antigens using a polyclonal specific serum according to previously described methods [11].

### Tissue DNA extraction and detection of *T. gondii* DNA by PCR

Tissue pieces from the placentomes that were taken for histological examination were also collected for parasite DNA detection by PCR. Samples of the liver from fetuses were also collected during necropsy and maintained at −20 °C for PCR analysis. Each PCR analysis was carried out in 5 pieces of different placentomes from each ewe and three different DNA extractions from each foetal tissue sample.

Genomic DNA was extracted from 20–50 mg of tissue using the commercial kit Maxwell® 16 Mouse Tail DNA Purification Kit, developed for automated Maxwell® 16 System (Promega, Wisconsin, USA) following the manufacturer’s recommendations. The concentration of DNA for all samples was determined by UV spectrophotometry and adjusted to 50–100 ng/μL. *T. gondii* DNA detection was carried out by a ITS1 PCR adapted to a single tube following procedures previously described [12]. Each PCR reaction was performed in a final volume of 25 μL using 5 μL of genomic DNA as template under PCR conditions as described [12]. Negative controls, including reactions without a template and DNA samples from non-infected foetuses were included in each round of DNA extraction and PCR. In each batch of amplifications, positive PCR controls with a quantity of *T. gondii* genomic DNA equivalent to 10 and 1 tachyzoites were also included. Positive controls were prepared by serial dilutions in a sheep genomic *T. gondii* PCR-negative DNA solution of 20 ng/μL. PCR products were visualized under UV light in 1.5% agarose/ethidium bromide gel. A reaction was determined as positive when a band of 227 bp was detected.

### Analysis of the peripheral humoral immune response

Sheep blood samples were collected from both groups at days −7, 0, 5, 11, 16, 21 and 25 pi by jugular venipuncture into 10 mL vacutainer tubes (Terumo Europe) without coagulant and allowed to clot. Serum was removed by centrifugation and samples were stored at −20 °C until they were used in an in-house indirect ELISA to measure anti *T. gondii* antibodies. Soluble antigens prepared from tachyzoites of ME49 isolate were used to coat 96 well microtiter plates. For this, 100 μL/well of antigen at 10 μg/mL diluted in carbonate buffer (63 mM, pH 9.6) was incubated overnight at 4 °C. Subsequently, non specific binding was blocked by adding 100 μL of bovine serum albumin diluted 0.05% in phosphate buffer saline (0.1 M, pH 7.6) containing 0.05% Tween 80 (PBST). After 2 h incubation at room temperature the plates were washed four times with PBST. Sera samples were diluted 1:100 in PBST and 100 μL of this dilution was added to each well and incubated for 60 min at room temperature. All samples were analysed in duplicate. After four washes in PBST, 100 μL of horseradish peroxidase conjugate protein G (Biorad, Hercules, USA) diluted 1:1500 in PBST was added as secondary Ab and incubated for 1 h at room temperature. Plates were washed as above before the addition of 100 μL per well of substrate ABTS (Sigma-Aldrich, Madrid, Spain) diluted 5.48 mg in 50 mL of citrate buffer 0.05 M, pH 4.0 with 0.0016% hydrogen peroxide. The reaction was stopped after 30 min at room temperature by the addition of 40 μL per well of a solution of hydrofluoric acid 0.1 M, and the OD read at 405 nm. In each plate, samples from the same positive and negative control sera were tested. The results were given as percent of positivity of the OD ration value (PP = [OD sample - OD negative control)/(OD positive control - OD negative control) × 100).

### Peripheral Blood Mononuclear Cells (PBMCs) stimulation assay and Interferon-gamma (IFN-γ) production analysis

Blood samples were collected from both groups at days −7, 0, 5, 11, 16, 21 and 25 pi by jugular venipuncture into 10 mL vacutainer tubes (Terumo Europe) with lithium heparin as anticoagulant. PBMCs were isolated by Ficoll gradient centrifugation following procedures previously described [13]. Once isolated, PBMCs were resuspended in RPMI 1640 medium (Gibco, Paisley, UK) supplemented with 10% fetal bovine serum (FBS; Lonza, Belgium) and 100X antibiotic/antimycotic solution (Santa Cruz Biotechnology Inc., CA, USA) to a concentration of 1 × 10^6^ cells/mL. The assays were performed in 24-well flat-bottom plates (Nunc, Roskilde, Denmark), with 5 × 10^5^ cells/well cultured with soluble antigen of *T. gondii* at a final concentration 2 μg/mL, concanavalin A (ConA, Santa Cruz Biotechnology Inc., CA, USA) at a final concentration of 5 μg/mL, or in medium alone. All cultures were performed in duplicate for each stimulus. Plates were cultured in a 5% CO_2_/37 °C/100% humidity atmosphere for 96 h. Thus, cell-free culture supernatants were stored at −70 °C for cytokine analyses.

Supernatant was assayed in duplicate for IFN-γ detection using a commercial bovine enzyme immunoassay kit (Bovigam; Prionics AG, Switzerland). Mean optical density (OD) for each experimental animal was calculated as the mean OD obtained from each supernatant from the *T. gondii* antigen stimulated cells divided by the mean OD of the same cells incubated with medium alone (negative control). Afterwards, mean OD was calculated for each experimental group. Supernatant from ConA stimulated cells were processed in a similar way as positive control for stimulation but not included in the analysis.

### Statistical analysis

Data of rectal temperature, IFN-γ and serological antibodies production were subjected to one-way analysis of variance using the general linear model procedure of SAS (SAS Inst. Inc., Cary, NC). The model included the fixed effect of the experimental treatment, where the animal was considered as the experimental unit. Logarithmic transformation of IFN-γ and serological antibody data was used to submit them to normal distribution. Analysis was performed for each sampling day and differences between means were determined with the Tukey test. Significance was declared at *P* < 0.05 unless otherwise indicated.

## Results

### Clinical observations

The most striking finding in the two groups was the occurrence of early abortions between days 7 and 11 pi.

In the first trial of experimental infections, according to the experimental design, six sheep (four infected and two controls) were culled from each group on day 5 pi, as scheduled (Table 1). These animals showed no evident clinical signs of disease, and all the foetuses from them were viable.

**Table 1:** Distribution of experimental animals according to the inoculated dose and dpi when culled or abortion occurred.

**Group**	**Challenge**	**No sheep per day^**				
		**5 dpi**	**7-11 dpi‡**	**12 dpi**	**21 dpi**	**Lambing**
A	2 × 10^3^†	4/2	11/0	1/2	0/0	0/4
B	5 × 10^2^	4/2	7/0	2/2	3/2	0/2

^: infected/control sheep; †: sporulated oocysts orally dosed; ‡: spontaneous abortions, the other timepoints correspond to serials culling of pregnant ewes.

Then, between days 7 and 11 pi, eleven sheep from group A (91.6% of the remaining infected sheep) aborted; one on day 7 and eleven between days 10 and 11. Then, the only remaining infected and not aborting sheep, plus two control animals, were culled on day 12 pi. The four control animals left delivered at full term and their lambs were culled 12 h after birth.

**Figure 1:**
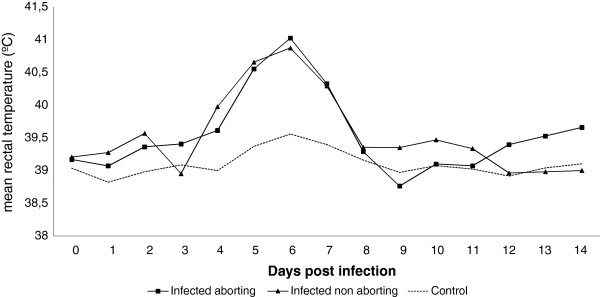
**Mean rectal temperature following *T. gondii *oocysts challenge.** Infected ewes, either showing abortion of not, showed elevation of rectal temperature between days 4 and 7 pi, with a peak of fever, over 40.5°, at day 6. Mean temperatures of control animals remained under 39.5° for the whole experiment.

After the occurrence of this high percentage of abortions in group A, the dose of infection for group B was dropped from 2 × 10^3^ to 5 × 10^2^ in a subsequent experimental infection. Similarly as in Group A, (four infected and two controls) were culled from each group on day 5 pi, as scheduled (Table 1). The infected sheep culled showed an elevation of rectal temperature at days 4 and 5 pi (Figure 1). Apart from this, no other evident clinical signs of disease were observed and all the foetuses from these animals were viable at the time of euthanasia. Then, in this group, seven sheep aborted (58.3% of the remaining infected sheep on the group) between days 9 and 11 pi; three on day 9, other three on day 10 and one on day 11. Afterwards, and according to the experimental design, two infected sheep, without any sign of disease, other than the transitory elevation of rectal temperature, were culled on day 12, along with two control animals. On day 21 pi, five sheep (three infected and two control), were culled, none of them having shown signs of disease other than transitory fever of the infected sheep (Figure 1). All the foetuses carried by the infected and control ewes culled on days 12 or 21 pi were viable at the time of euthanasia. Finally, two non infected ewes were left to deliver at the end of their pregnancy. Their lambs were culled 12 h after birth.

When analysing the temperature of animals from group B, statistically significant differences in the mean rectal temperature were found between infected and control sheep at days 2, 5 and 6 pi (*P* < 0.001). The mean temperature of the infected group was higher from days 4 to 7 pi, with a peak of 41.3 °C on day 6 pi. When analysing groups of aborting and non aborting animals within the infected sheep, no significant differences were found between them (Figure 1).

### Gross and microscopic lesions

#### Infected ewes showing early spontaneous abortion

From all the sheep that suffered abortion, only eight (four from group A and four from group B) were examined. The remaining placentas were too autolytic to be properly evaluated. These eight placentas and all the aborted foetuses had the same gross and microscopic findings, regardless of the experimental group (Table 2).

**Table 2:** **Percentage of studied cases showing histological lesions and/or *T. gondii *** **DNA identification according to the inoculated dose and dpi when culled or abortion occurred.**

**Group**	**Challenge**	**% of positives out of the studied cases^**									
		**5 dpi**		**7–11 dpi‡**		**12 dpi**		**21 dpi**		**Lambing**	
		**HE**	**PCR**	**HE**	**PCR**	**HE**	**PCR**	**HE**	**PCR**	**HE**	**PCR**
A	2 × 10^3^†	0/0	0/0	100/9	0/0	0/0	100/0	ns	ns	0/0	0/0
B	5 × 10^2^	0/0	0/0	100/63	33/0	0/0	100/0	§100/75	100/100	0/0	0/0
Total		0/0	0/0	100/32	25/0	0/0	100/0	§100/75	100/100	0/0	0/0

^: numerator: percentage of placentas showing lesions/*T. gondii* DNA identification, denominator: percentages of foetuses showing lesions/*T. gondii* DNA identification; †: sporulated oocysts orally dosed; ‡: spontaneous abortions, the other timepoints correspond to serials culling of pregnant ewes; §: These were lesions of “classical” toxoplasmosis.

The placentas showed a dark red surface. The amniotic liquid was turbid and the foetuses, none of them viable, showed variable degree of autolysis and oedema.

**Figure 2:**
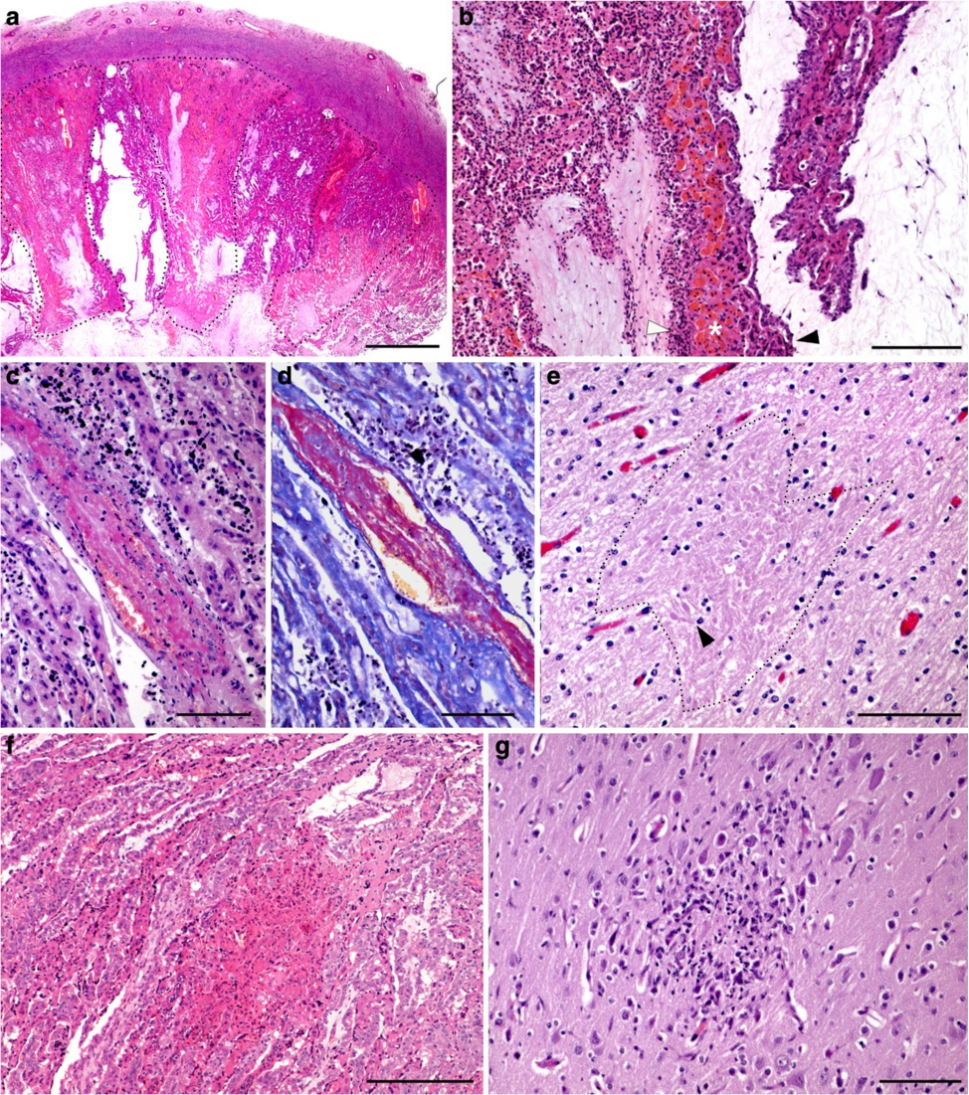
**Histological lesions of placenta and foetuses from infected ewes. a**: Coronal section of a placentome from an acute case abortion. Several triangular areas of coagulative necrosis, involving both caruncular and cotyledonary villi (dotted line). HE. Bar = 2 mm. **b**: Thrombosis in the placenta of an acute abortion case. The thrombus is partially occluding the lumen of the vessel in the caruncular septa. HE. Bar = 100 μm. **c**: The presence of fibrin forming the thrombus is shown by the red staining in a serial section from the same placentome shown in **b**. MSB. Bar = 100 μm. **d**: Placentome from an acute abortion case. Detail from the margin of an infarct. The picture is divided in two areas by a caruncular villi showing congestion of the septal vessels (asterisk). At the left side there is abundant cellular debris between the denuded caruncular and foetal villi (white arrowhead). Caruncular villi in this area are necrotic, while cotyledonary villi show mesenchymal hyalinization. Note the relatively spare cotyledonary villus at the right side of the picture, with clear mesenchyme and intact trophoblasta layer (black arrowhead), and also the caruncular villus at the right superior corner with no evident lesions. HE. Bar = 200 μm. **e**: Area of leukomalacia (dotted line) in the white matter of a foetal brain from an acute phase abortion. Note numerous axonal spheroids (arrowhead) within the focus of malacia. HE. Bar = 100 μm. **f**: Necrotic in the placentome of an infected ewe culled at 21 dpi. The lesion affects both caruncular and cotyledonary villi. Bar = 25 μm. **g**: Focus of coagulative necrosis in the foetal white matter from an infected ewe culled at day 21 pi. Note the abundant infiltration of glial cells surrounding the necrotic focus. HE. Bar = 100 μm.

Histologically, few multifocal to coalescent areas of coagulative necrosis surrounded by a marked vascular congestion, consistent with infarcts, were observed in the placentomes. These areas were triangular in shape with the base at the endometrial stalk of the placentome (Figure 2a) and involved a discrete group of maternal villi. They generally occupied the mid region of the placentome or could extend to the arcade or chorionic plate.

In the areas of infarct, the stroma of the caruncular villi appeared highly eosinophilic and amorphous, with scant chromatin debris from necrotic cells. These villi were denuded of epithelial cells, which were desquamated and appeared as eosinophilic, necrotic debris between the remnants of caruncular and cotyledonary villi (Figure 2b). Within the septa, the vessels were congested and dilated. Inside some of the vessels of this area, thrombi composed of masses of a hyaline amorphous material stained positive for fibrin by MSB technique and containing interspersed blood cells, were identified (Figures 2c and d). The foetal villi that were located in the vicinity of the necrotic caruncular villi showed hyalinization of the mesenchyme (more evident in those from group A, infected at 90 days of gestation, when the mesenchyme is more prominent) and necrosis of the throphoblasts (Figure 2b). The latter suffered a variety of degenerative changes, ranging from cytoplasmic eosinophilia shrinkage, nuclear pyknosis and karyorrhexis, to complete detachment from the foetal villi, forming isles of necrotic debris together with the remnants of epithelial maternal cells.

The studied foetuses also showed a variable degree of autolysis that hampered the sampling procedure, especially in those foetuses already expelled when found. In most of these cases, no more than one slide with unrecognizable areas of the brain was available for histological examination. Two of the twenty five examined foetuses were mummified. Histological examination showed microscopic lesions in only eight out of the twenty five foetuses (Table 2). They were located in the brain and characterized by scarce foci of coagulative necrosis and discrete axonal swelling in the periventricular area (Figure 2e), and generalized congestion of the white matter vessels. Besides the foci of leukomalacia, one of these foetuses showed numerous, minute haemorrhages in the white matter. No other brain lesions were found in the rest of the foetuses.

No parasite was detected in any of the sections from the placenta or the foetal brain when the immunohistochemical technique was applied.

#### Non-aborted infected ewes culled according to schedule

No gross or microscopic lesions were found in the placentas or foetuses of any of the animals culled on day 5 pi (eight sheep in total, four from each group), or on day 12 (one from group A and two from group B, Table 2).

The placentas and three out of four foetuses from the three sheep culled on day 21 pi from group B showed lesions compatible with those described classically as associated with ovine toxoplasmosis (Table 2). In the placenta, they were characterized by a mild multifocal necrotic placentitis denoted by several well-delimited areas of coagulative necrosis in the septum of caruncular villi with absence of evident inflammatory cell infiltration (Figure 2f). No vascular changes were detected in any case.

While no parasites were recognised under HE staining, numerous parasitophorous vacuoles were evident inside this foci when section were immunolabeled against *T. gondii* antigen.

Affected foetuses showed a multifocal non-purulent hepatitis characterized by several randomly distributed foci of lymphocytes and macrophages; occasionally, they were surrounding a small area of central necrosis. This inflammatory infiltrate was also present in the periportal spaces of the liver. Two out of these foetuses also showed a perivascular infiltration of mononuclear cells in the lung. Few glial foci with a central area of necrosis were detected in the brain of one foetus (Figure 2g). Immunohistochemical labelling of these samples showed the presence of few parasitophorus vacuoles within the necrotic area of the brain and liver in the three foetuses.

#### Control ewes

No gross or microscopic lesions were found in any of the placentas or foetuses from the control ewes studied.

### Identification of *T. gondii* DNA in tissue samples

Tissue samples from the placentomes of four aborted ewes (one from group A and three from group B) were analysed by PCR for parasite detection. Besides, placentome samples from further five non aborting ewes (three infected and two control) were also processed for PCR analysis: one from group A, culled at day 12 pi; two from group B euthanized at 12 and 21 dpi respectively and one uninfected control animal from each group.

As shown in Table 2, toxoplasma specific DNA was detected in the placentomes from one ewe (Group B, aborted on day 9 pi) out of four aborted dams analysed (25%), and in two out of the three infected non aborted ewes examined (66.6% of the examined), one studied at day 12 pi (group A) and the other culled at day 21 pi (group B). No parasite DNA was identified in the placentas from the control sheep studied.

Liver samples from aborted foetuses (eleven from Group A and nine from Group B), foetuses from infected and non aborted ewes (one from group A, culled at 12 dpi, and three from Group B, two culled at day 12 pi and one at day 21 pi) and foetuses from non infected dams (two, one from each group, culled at day 12 pi) were processed for molecular detection of *T. gondii*.

The liver samples from the twenty aborted foetuses were all negative (Table 2). Regarding the foetuses from infected non aborted ewes, only the foetal liver from one sheep culled at day 21 pi (Group B) was positive for *T. gondii* DNA while no parasite was found in the remaining five foetuses studied. The two foetuses from control ewes were also negative (Table 2).

### Serological antibodies against *T. gondii*

**Figure 3:**
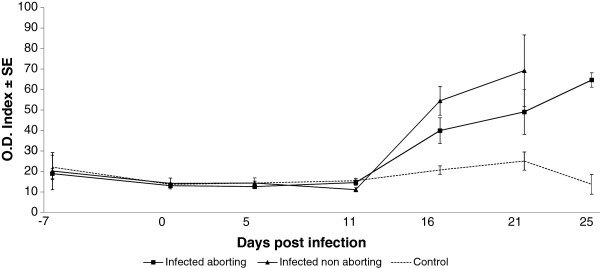
**Kinetics of the antibody production in infected and control ewes.** Infected animals showed an increase on serological antibodies from day 16 pi. Differences between ewes suffering or not acute phase abortions were not significant.

Figure 3 shows the Ab production in the different experimental animals. With the purpose of analysing the relationship between the presence of serological Ab against *T. gondii* and the occurrence of acute phase abortions, experimental ewes have been grouped regardless of the time of gestation when they were infected, into the following three groups: sheep suffering abortion, infected and non aborting ewes (culled at day 5 pi -Groups A and B- and days 12 and 21 pi -only Group B) that had not suffered abortion, and finally a control group made up of all the non infected sheep. It is noteworthy that ten, out of eighteen aborted sheep, were not culled and remained alive for the duration of the experiments, so they were sampled for γ-IFN production and serological antibodies assays. In any case, no significant differences were observed in the kinetic of serological antibodies when comparing the infected sheep from group A and B.

All infected ewes, regardless of the occurrence or not of abortion, showed a sudden increase in the O.D. value at 16 dpi that was progressively increasing until the end of sampling. At days 16, 21 and 26 pi, the O.D. of the infected sheep (aborting and non aborting) was significantly higher (*p* < 0.01 on days 16 and 21 pi, and *p* < 0.001 on day 26 pi) than the control sheep. Although the aborting ewes showed a lower mean O.D. value than the infected non aborting sheep on days 16 and 21 pi, this difference was not statistically significant.

### IFN-γ production analysis

**Figure 4:**
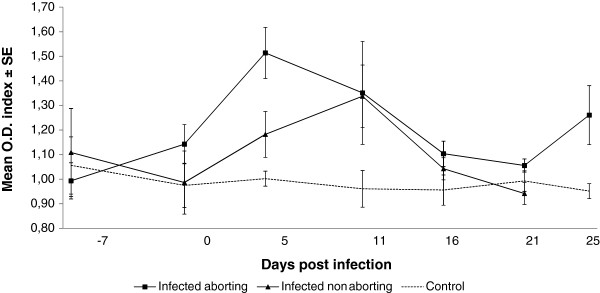
**Kinetics of the specific IFN-γ release by PBMC stimulation assay against *T. gondii *antigen.** Although differences between ewes suffering or not acute phase abortions were not significant, the peak of IFN-γ release appeared sooner in the former than in the latter. Values of the O.D. index in the control group remained low over the whole experiment.

Similarly as with the analysis of the serological response, γ-IFN production by PBMCs was analysed grouping data according to the occurrence of abortion or not on sheep and a control group. All infected sheep showed a weak increase in the production of IFN-γ (< 2.5 fold-change over basal levels). However, there was a significant (*p* < 0.001) increase in IFN-γ production at day 5 pi of the aborting sheep when compared to the control group, while the mean OD index of the infected non aborting sheep was in between both groups. A non significant increase in IFN-γ production was observed in the infected but non aborting ewes when compared to the control group at day 11 pi (Figure 4).

## Discussion

In this study we analyse the main clinical, pathological, parasitological and immunological findings associated with the occurrence of numerous acute phase abortions in experimentally infected sheep after oral inoculation with 2000 or 500 sporulated oocysts of *T. gondii* M4 isolate. The occurrence of acute phase abortions in ovine toxoplasmosis has been previously reported in a few experimental studies [7,8]. Similarly to those descriptions, the ewes suffering acute phase abortion in the current study had no detectable serological antibodies against *T*. gondii at the time of abortion nor foetal or placental lesions associated with the presence of *T. gondii*. None of the placentas or foetuses from aborting ewes showed *T. gondii* antigen either. Furthermore, parasite DNA detection was limited to some placentomes from one out of eight aborting sheep and it was undetected in foetal samples. All these features are different from what is regularly found in “classical” toxoplasmosis abortions. This has serious implication for the diagnosis of the disease in field cases of reproductive failure, as samples usually taken when the abortion occurs, generally foetus, foetal membranes and maternal serum, would be negative to parasite DNA or specific antibodies detection.

The pathogenesis of the abortion in ovine toxoplasmosis is not yet well understood [1] but it seems to require the presence of the parasite in the placenta. It is thought that abortion in early pregnancy is a consequence of the damage caused by the replication of the parasite in foetal tissues [5] or due to immunological imbalance in the maternal-foetal interface [2], while foetal death in late gestation would be the consequence of lack of placental function due to extensive necrotic lesions in the placentomes caused by the parasite [14]. However, in previous descriptions of acute phase abortions, parasite DNA was not found in the placentas or foetal samples [7,8]. The improved sensitivity of the ITS-nested PCR technique used in the current analysis, compared to previous studies, may have allowed the identification of *T. gondii* DNA in one out of four placentas analysed from aborting ewes. The scarcity of positive identification by PCR, plus the absence of parasite antigen in the placentomes, may suggest that the replication of the parasite in placenta or foetus was not the direct cause of these abortions. In previous studies, it was suggested that the pyrexia developed after the infection could have been the trigger for those abortions, although no specific mechanism for their occurrence was elucidated [7,8]. It has been shown that hyperthermia from febrile infections can result in abortions or, when the temperature elevation is not too high, in birth defects mainly affecting the central nervous system [15]. In the present study, those infected sheep not suffering abortion showed a similar curve of body temperature than ewes having acute phase abortions, reaching more than 41 °C degrees at the peak of the fever episode, with no significant differences in the temperature between both infected groups. In none of the cases were malformations of the CNS noticed during post mortem analysis. Furthermore, in numerous studies where pregnant sheep were also orally infected with sporulated oocysts and showed similar episodes of fever, developing body temperatures higher than 41°, the occurrence of acute phase abortions was not described in any of them [5,16-18]. According to these results, it can be concluded that hyperthermia alone is unlikely to be the cause of acute phase abortions in toxoplasmosis.

The infectious inoculum, either the dose, the viability of the inoculated oocysts or the virulence of the used isolate, could have played a role in the triggering of abortions. The dose administered at day 90 of gestation (2000 sporulated oocysts), has been previously used in several studies without the occurrence of acute phase abortions [16,18-20]; however, acute phase abortions were recorded in other studies using the same dose [7,8]. A recent study employed a higher dose of oocysts (3000) of the same isolate (M4) without finding any acute phase abortion [21], also suggesting that the isolate used in this experimental inoculation was not especially virulent. In the current study, the dose used at day 120 of gestation in group B, which was dropped to 500 oocysts, caused a significant reduction in the number of acute abortions in comparison with the 2000 oocysts administered to group A (91.6% vs. 58.3%). Both results are striking not only due to the lack of occurrence of acute phase abortions using 2000 oocysts in previous works [16,18-21], but also because a dose as low as 500 oocysts induced such high rate of them at the last term of pregnancy, suggesting that the dose inoculated is playing a role in the occurrence of acute phase abortions. In our study, sporulated oocysts were used shortly after their shedding from cats, within a month. It seems then feasible to speculate that the viability of these oocysts was extremely high and this could be the cause of an overwhelming exposure of the pregnant ewes to a high number of parasites at one point. It has been suggested that significant variability in the viability of oocysts could be the cause of different findings in experimental studies using similar doses, so the verification of the infectivity by bioassay was recommended as a way to standardized protocols [1]. A dose of 100 oocysts from the batch used in the current experiment caused 100% mortality 4 weeks after infection of mice, Porton strain (Frank Katzer, data not shown).

On the other hand, the participation of the virulence of the isolate in the occurrence of the abortions should also be considered. It has been described that the continuous passage of toxoplasma in mice could enhance its virulence [22]. Because the oocysts used in the current experiment were from an isolate (M4) that cannot be considered a strain, there is a possibility that there has been a selection towards high virulence within the isolate during the successive passages, especially because of the sulphadimidine treatment. This sulfonamide would mitigate the effect of the more aggressive variants within the isolate and helped to maintain them without being lethal to the mice. The increase of virulence of the isolate is sudden, occurring from one passage to another [22], which may explain why previous infections with the same isolate were not associated with acute abortions [21] while causing them in the current study after a limited number of passages.

The gestational age, and not only the reduction of the infectious dose, could have also influenced in the variation of abortion rate between both groups. The period of gestation when infection occurs is a key factor which determines the consequences of ovine toxoplasmosis. While abortion is the most common consequence after infection in midgestation, viable congenitally infected lambs could be born when it occurs later [2]. Also, the breed of the sheep may be another factor involved in the high number of acute phase abortions found in this study. It has been suggested that certain breeds, such as Charollais, are more susceptible to suffer abortions caused by *T. gondii* infection than Suffolk sheep [23]. It might be the case that Churra breed is more susceptible to develop acute phase abortions than other breeds when infected by *T. gondii*. However, toxoplasmosis is a common disease among sheep in our region and practitioners or farmers with Churra flocks have not observed any abnormal susceptibility of this species to suffer from abortions, either from *T. gondii* or any other abortifacient agent. The implication of these two variables, gestational age and breed, in the pathogenesis of acute phase abortions deserves further investigation.

None of the studied placentas or fetuses from ewes suffering acute phase abortion showed typical lesions of toxoplasmosis, in opposition to those ewes culled at 21 dpi. Instead, all placentomes from acute phase abortions showed infarcts and thrombosis in the caruncular septum. Besides, a number of fetuses from these episodes showed periventricular leukomalacia, a lesion already associated with foetuses suffering hypoxia at midgestation [24]. These pathological findings strongly suggest a different pathogenic mechanism in the development of abortion between acute phase and classical toxoplasmosis.

Thrombosis and associated ischemic necrosis (infarcts) in the caruncle are common lesions related to abortion in ruminants. They are mainly due to mycotic and few bacterial infections of the placenta that cause severe vasculitis characterized by abundant infiltration of inflammatory cells [25]. Such infiltration was absent in all the studied cases of acute phase abortions, so the pathogenesis of the thrombosis is thought to be triggered by mechanisms other than the inflammation of the vascular wall. *T. gondii* tachyzoites can replicate in a broad range of cell types such as endothelial cell [3], and thrombosis due to the direct damage of endothelial cells provoked by tachyzoite replication in these cells has been described in immunosupressed patients suffering human acquired immunodefiency syndrome [26,27] or elderly dogs with underlying diseases causing immunosupression [28]. However, in those cases, *T. gondii* tachyzoites were numerous in the lesions and readily demonstrable by immunohistochemichal labeling [27,28]. In the current study, the absence of parasite antigen in all the placentomes from the abortion cases, plus the very low frequency of detection of parasite DNA by the highly sensitive ITS- nested PCR, suggest that the cause for thrombosis, and thus abortion, was other than the direct damage of the endothelium by the parasite.

It has been demonstrated that murine trophoblasts secrete fibrinogen-like protein 2 (fgl2), a prothrombotic factor that favors fibrin deposition and thrombosis, in response to proinflamatory cytokines, such as IFN-γ or TNF-α [29]. *T. gondii* infection is known to trigger the secretion of IFN-γ in sheep [30] and mice [31]. Furthermore, it has been recently shown that the mere inoculation of secretion antigens from toxoplasma causes elevation of IFN-γ serum levels and abortion in mice, with no need of parasite replication [32]. Bearing in mind that these results are from different experimental models, it is tempting to hypothesize that fgl2 might be playing a role in favoring the occurrence of placental thrombosis found in these acute phase abortions, being its secretion related to an increase of IFN- γ levels that could be consequence of the infectious dose. In the present study, all infected sheep showed secretion of IFN-γ in the lymphocyte stimulation assays soon after infection, but in the group of sheep suffering acute phase abortions, this peak of secretion appeared one week before those not suffering abortion. The relation between IFN-γ secretion both at peripheral or local levels, the occurrence of these abortions, and its possible relation to FGL2 expression, deserves further investigation.

In conclusion, this study describes, and analyzes, the occurrence of acute phase abortions during an experimental infection of ovine toxoplasmosis. The microscopic lesions found in the placenta and foetuses from aborted ewes suggest the involvement of pathogenic mechanisms different from those proposed for classical toxoplasmosis, where the abortion would be consequence of the damage caused by parasite replication in the placenta or foetus [2,5,14]. The occurrence of thrombosis and infarcts in the placenta, without the necessary presence of the parasite in relation to the lesions, suggests that the acute phase abortions in toxoplasmosis could be the consequence of the vascular damage caused by mechanisms other than parasite replication.

## Competing interests

The authors declare that they have no competing interests.

## Authors’ contributions

PC performed the experiment, participated in all the immune response and pathological studies and collaborated in the analysis of the data and writing the paper. MFue contributed to the sample collection, pathological studies and interpretation of the results. IF prepared the infectious inocula and collaborated in writing the paper. MFer participated in the sample collection and pathological studies. MCF participated in the sample collection pathological studies and collaborated in the manuscript preparation. JMG participated in preparation of the infectious inocula. CGL collaborated in the immunological studies and sample collection. FK collaborated in the design of the experiment and helped to draft the manuscript as wells as to the preparation of the infectious inocula. JRC collaborated in the molecular analysis of the samples and helped to writing the manuscript. LMOM contributed to the design and supervision of the experiment and helped to draft the manuscript. VP collaborated in the designs, development and supervision of the experiment, participated in all the pathological studies, analysis of the data and writing the paper. JB conceived and designed the experiment, participated in the pathological studies, analysed the data and wrote the paper. All the authors read and approved the final manuscript.
